# Theoretical and methodological considerations in evaluating large-scale health information technology change programmes

**DOI:** 10.1186/s12913-020-05355-7

**Published:** 2020-05-27

**Authors:** Kathrin Cresswell, Aziz Sheikh, Bryony Dean Franklin, Marta Krasuska, Hung The Nguyen, Susan Hinder, Wendy Lane, Hajar Mozaffar, Kathy Mason, Sally Eason, Henry W. W. Potts, Robin Williams

**Affiliations:** 1 Usher Institute, The University of Edinburgh, Edinburgh, UK; 2 University College London School of Pharmacy, London, UK; 3 NIHR Imperial Patient Safety Translational Research Centre, London, UK; 4 Institute for the Study of Science, Technology and Innovation, The University of Edinburgh, Edinburgh, UK; 5 National Health Services Arden and Greater East Midlands Commissioning Support Unit, Warwick, UK; 6 Business School, The University of Edinburgh, Edinburgh, UK; 7 University College London Institute of Health Informatics, London, UK

**Keywords:** Health information technology, Implementation, Evaluation

## Abstract

**Background:**

Attempts to achieve digital transformation across the health service have stimulated increasingly large-scale and more complex change programmes. These encompass a growing range of functions in multiple locations across the system and may take place over extended timeframes. This calls for new approaches to evaluate these programmes.

**Main body:**

Drawing on over a decade of conducting formative and summative evaluations of health information technologies, we here build on previous work detailing evaluation challenges and ways to tackle these. Important considerations include changing organisational, economic, political, vendor and markets necessitating tracing of evolving networks, relationships, and processes; exploring mechanisms of spread; and studying selected settings in depth to understand local tensions and priorities.

**Conclusions:**

Decision-makers need to recognise that formative evaluations, if built on solid theoretical and methodological foundations, can help to mitigate risks and help to ensure that programmes have maximum chances of success.

## Background

Many countries worldwide see large-scale system-wide health information technology (HIT) programmes as a means to tackle existing health and care challenges [1–3]. For example, the United States (US) federal government’s estimated $30 billion national stimulus package promotes the adoption of electronic health records (EHRs) through the Health Information Technology for Economic and Clinical Health (HITECH) Act [4]. Similarly, the English National Health Service (NHS) has invested £4 billion in a national digitisation fund [5]. Digitisation strategies and funding schemes reflect national circumstances, but such programmes face common challenges. These include for example tensions in reconciling national and local requirements. While some standardisation of data transactions and formats is essential to ensure interoperability and information exchange, there is also a need to cater for local exigencies, practices and priorities [6].

Summative evaluations that seek to capture the eventual outcomes of large national programmes appear to answer questions about the effectiveness of public investments. However, funders and administrators are under pressure to demonstrate outcomes quickly - often within the lifetime of programmes, whilst the full benefits of major change programmes can take a long time to materialise. Premature summative evaluation can generate unwarranted narratives of “failure” with damaging political consequences [7].

The success or failure of HIT projects involves many different dimensions and at times incommensurable factors [8, 9]. The political context may change within the medium- to long-term timeframes of a major change programme, [6, 8] as seen with some aspects of the English National Programme for Information Technology (NPfIT) [10, 11]. A formative evaluation approach cannot avoid these issues, but can help to better navigate the associated complexities. It can identify apparently productive processes, emerging unintended consequences, and inform the programme’s delivery strategy in real time [12, 13]. It seeks to capture perceptions of actors involved about what is, and is not, working well and feed back findings into programme management. Such evaluations often involve gathering qualitative and quantitative data from various stakeholders and then feeding back emerging issues to implementers and decision-makers so that strategies can be put in place to mitigate risks and maximise benefits.

Our team has conducted several formative evaluations of large HIT programmes and developed significant expertise over the years [14–16]. In doing so, we have encountered numerous theoretical and methodological challenges. We here build on a previous paper discussing the use of formative approaches for the evaluation of specific technology implementations in the context of shifting political and economic landscapes [10, 14]. In this previous work (Table 1), we described the complex processes of major HIT implementation and configuration. We argued that evaluation requires a sociotechnical approach and advocated multi-site studies exploring processes over extended timeframes, as such processes are not amenable to conventional positivist evaluation methodologies.

**Table 1:** Summary of recommendations for formative evaluation of large-scale health information technology [17]

Before-during-after study designs are ill suited to explore large-scale electronic health record implementations due to shifting policy landscapes and over-optimistic deployment schedules. They also do not sufficiently take local views and interpretations into account.	
Formative evaluations need to consider this changing landscape and explore stakeholder perspectives to gain insights into how local actors understand and implement change.	
Sociotechnical approaches can help to conceptualise the interactions between people, technology and work processes. They can help to draw a more nuanced picture of the implementation and adoption landscape than traditional positivist paradigms.	

We here offer an extension of this work to explore not only implementations of specific functionality (such as electronic health records (EHRs)), but their programmatic integration with ancillary systems (e.g. electronic prescribing and medicines administration, radiology). This can help to gain insights into the emergence and evolution of information infrastructures (systems of systems) that are increasingly salient as we see functional integration within hospitals and across care settings. We also consider mechanisms of spread, evolving networks/processes, and vendor markets [17].

## Main text

### The difficulty of attributing outcomes

The first challenge concerns the difficulty of attributing outcomes (i.e. exploring what caused a specific outcome) for major changes in HIT. Although often required to justify investments, the direct effects of complex HIT such as EHRs are difficult to track and measure [18]. This is particularly true for large-scale transformative and systemic upgrades in infrastructures, which are not one-off events, but occur through multiple iterations and interlinkages with existing systems. Such systems tend to have distributed effects with hard-to-establish gradually emerging baselines (when compared to local discrete technologies implemented in specific settings, although the effects of these can also be hard to measure) [19]. Infrastructure renewal is a long term process where current achievements rest on earlier upgrades over long timeframes as systems are incrementally extended and optimised [20]. An example may be the implementation of EHRs and their integration with ancillary systems. Here, decision-makers are championing not just one, but multiple implementations of various transformative systems.

Theoretically informed formative evaluations that draw on science and technology studies and acknowledge the interrelationship between social and technological factors can help to address this issue [21]. A particularly effective methodology is exploring selected settings in depth to understand local complexities, while also monitoring a wider number of settings in less detail to understand general trends. Complex research designs drawing on case study methods and a range of sociotechnical approaches can help to explore how technological and social factors shape each other over time [22]. They can therefore provide an insight into local changes and potential mechanisms leading to outcomes [22]. In our current work on evaluating the Global Digital Exemplar Programme, for example, we are conducting 12 in-depth case studies of purposefully recruited hospitals. In addition, we are collecting more limited longitudinal qualitative data across all 33 hospitals participating in the Programme [23]. This research design offers a balance between depth (achieved through the case studies) and breadth (achieved through testing emerging findings across the broader sample).

### Balancing local diversity and autonomy with national aims

Decision-makers cannot simply roll out standard solutions across the health service as sites vary in terms of clinical practices, existing information systems and data structures, size and organisational structures, contexts and local demographics. A key challenge for evaluation of large programmes is reconciling tensions between bringing specific sites up to international best practice, and levelling up the local ecosystem [24]. Organisational settings differ in their local contexts, structures and (emerging) service configurations. They are often separate autonomous entities that may be in competition [25]. Various groups of clinical staff and decision-makers may have different priorities (e.g. between decision-makers and various groups of clinical staff). Programme visions may be differently interpreted by local stakeholders, which can lead to unanticipated outcomes and deviation from central aims. In the US, the Meaningful Use criteria have for instance resulted in increasing implementation of EHRs, but the impact on quality and safety is still unknown and concern has been expressed that they may have stifled local innovation [26].

**Fig. 1:**
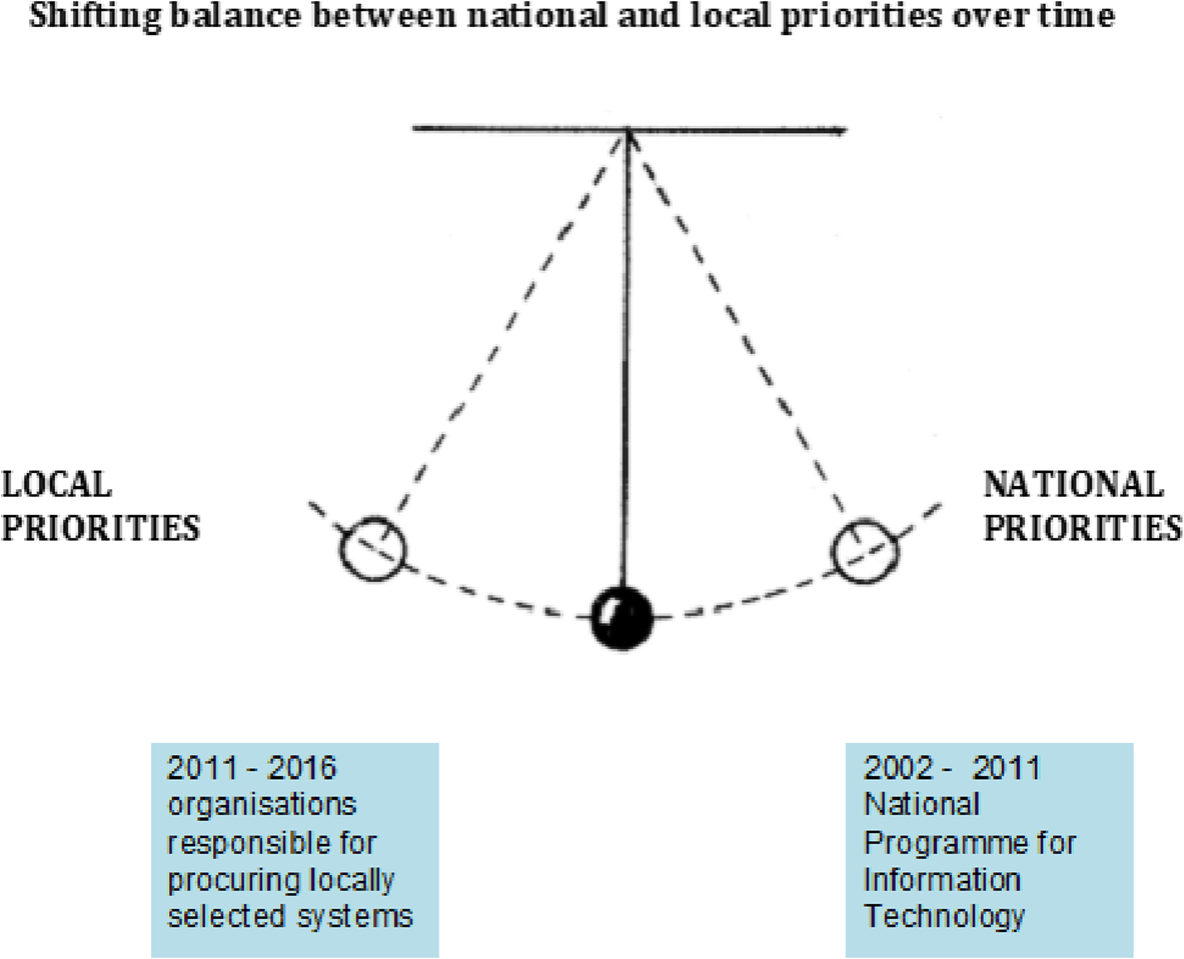
Tension between local and national priorities in large health information technology programmes [3]

There is a tension between local and national priorities – and there is no stable way to reconcile these. Instead, strategies constantly shift between these poles, never standing still, pulled by a network of stakeholder groups with conflicting interests in a process that has been conceptualised as a swinging pendulum (Fig. 1) [14]. For example the UK NPfIT exemplified a strong pull towards national priorities, with a strategy that focused on concerted procurement and interoperability. In the period that followed, organisations were responsible for the procurement of locally selected systems. The pendulum swung the other way.

To explore this process and associated tensions, evaluators need to study evolving networks, relationships, and processes to understand how various stakeholders are mobilised nationally and locally as part of the change programme and what the perceived effects of these mobilisations are. This may involve working closely with national Programme Leads to identify current policy directions and intended national strategy, whilst also exploring local experiences of this strategy. From our experience, it can be helpful to move from arms-length critical analyst to constructive engagement with different stakeholders groups. Establishing long-term personal relationships with senior decision-makers whilst retaining independence is important is this respect. These need to be characterised by mutual trust and frank discussion where evaluators play the role of a ‘critical friend’ at times delivering painful truths.

A recurring example of tensions in our work relates to progress measures. National measures of progress designed to provide justification for programme resources are liable to clash with local priorities and circumstances. Participating local organisations may negatively perceive achieving these as requiring large amounts of resources in comparison to limited local benefits and driven by the need to satisfy reporting demands. Some agreement over a limited core set of measures to satisfy both local and national demands may be helpful.

### The evolving nature of HIT programmes over time

Takian and colleagues noted how the policy context changed in the course of a single long-term change programme [17]. These factors may result in various stakeholders chasing moving targets and scope creep. For example, the economic recession of 2008–13 heavily influenced the English NPfIT, which led to a lack of sustained funding [27].

Although important, shifting socio-political environments only constitute part of the picture. A long-term view of nurturing evolving infrastructures highlights that visions of best practices will inevitably change over time [28]. They also often have no definite end point and there is at times no consensus about strategic direction. We have previously discussed this in the context of digital maturity, which is a somewhat contested concept [29]. Different kinds of programme management and evaluation tools may be needed that give cognisance to this kind of evolution. These may include an emphasis on flexibility and reflexivity, where decision-makers can adjust strategies and roadmaps in line with emerging needs and changing environments. This approach will also require learning historical lessons and drawing on the wealth of experience of those who have experienced similar initiatives first hand.

Changes in medical techniques and diagnosis, models for care delivery, and vendor offerings affect available technologies (and vice versa). The market may not immediately be able to respond to new policy-driven models, and therefore evaluations and policies need to consider these dimensions [30]. This may involve exploring evolving vendor-user relationships, the emergence and mobilisation of user groups, procurement frameworks, and market diversity [31]. Our work, for instance, shows that, reinforced by the English NPfIT, multi-national mega-suite solutions revolving around core EHR systems increasingly dominate the UK market. These offer a relatively well-established and reliable pathway to achieving digital maturity and interoperability. The alternative pathway involves knitting together EHRs with a range of other functionality provided by diverse vendors. This may offer advantages in allowing an adopter to achieve a Best-of-Breed (BoB) solution unique to each local setting, and potentially better suited to local organisations [32]. However, there are difficulties for vendors of modular solutions designed for BoB to enter the market and develop interfaces. Existing EHR vendors are struggling to upgrade their systems to become mega-packages. Implementers must carefully consider interoperability challenges and innovation opportunities afforded by various systems. Programmes must ensure procurement approaches stimulate (or at least to not inhibit) a vibrant marketplace.

### Scaling of change through developing a self-sustaining learning ecosystem

Large HIT change programmes are often concerned with not only stimulating local changes but also with promoting ongoing change ensuring that efforts are sustained and scaled beyond the life of the programme [33]. But this is not straightforward, partly due to lack of agreement over suitable metrics of success and partly due to limited understanding of the innovation process [34].

Studies of the emergence and evolution of information infrastructures have in turn helped articulate new strategies for promoting/sustaining such change [35–37]. However, the notion of scaling-up tacitly implies that innovation stops when diffusion starts. A more nuanced perspective flags that innovations evolve as they scale (‘innofusion’), requiring strong learning channels between adopter communities and vendors [38].

Evaluators can explore success factors and barriers to scaling qualitatively and formatively feed these back to decision-makers who can then adjust their strategies accordingly. Evaluation needs to address local change in tandem with evolving networks at ecosystem level. By studying a range of adopter sites and their relationships with each other, as well as other stakeholders that are part of the developing ecosystem, evaluators can identify mechanisms that promote digital transformation and spread. Understanding these dynamics can also help decision makers focus strategy on achieving programme objectives. By addressing networks and relationships, evaluators can, for example, explore how knowledge spreads throughout the wider health and care ecosystem in which the change programme is embedded, and how stakeholders were motivated to exchange and trade knowledge [39].

## Conclusions

We are now entering an era that emphasises patient-centred care and data integration across primary, secondary and social care. This is linked to a shift from discrete technological changes to systemic long-term infrastructural change associated with large national/regional HIT change programmes. There are some attempts to characterise and study these changes including our own [17]. However, these provide only a partial picture, which we have built on here based on our ongoing experiences reflecting our current thinking (see Table 2).

**Table 2:** Summary of key recommendations emerging for evaluating large-scale health information technology change programmes

Study selected settings in depth in order to understand local complexities, whilst also exploring the wider number of settings that are part of the change programme to understand general trends	
Study evolving networks, relationships, and processes exploring how various stakeholders are mobilised nationally and locally as part of the change programme, and the perceived effects of these mobilisations	
Study changing organisational, economic, political, vendor and market contexts	
Study mechanisms of spread to accelerate programme objectives and align strategy accordingly to focus on these opportunities	

We now need new methods of programme management geared towards developing learning in ecosystems of adopters and vendors. These evolutionary perspectives also call for broader approaches to complex formative evaluations that can support the success of programmes and help to mitigate potential risks.

Although there is no prescriptive way to conduct such work, we hope that this paper helps decision-makers to commission work that is well suited to the subject of study, and implementers embarking on the evaluative journey to navigate this complex landscape.

## Data Availability

Not applicable.

## Abbreviations

BoB: Best-of-Breed
EHR: Electronic Health Record
HIT: Health Information Technology
HITECH: Health Information Technology for Economic and Clinical Health
NHS: National Health Service
NPfIT: National Programme for Information Technology
US: United States
